# FAMILY CAREGIVING AMONG AFRICAN AMERICANS AND BLACK IMMIGRANTS: UNDERSTANDING DIABETES MANAGEMENT CHALLENGES

**DOI:** 10.1093/geroni/igae098.0454

**Published:** 2024-12-31

**Authors:** Dorothy Addo-Mensah, Jamie Conklin

**Affiliations:** University of North Carolina, Chapel Hill, North Carolina, United States; University of North Carolina, Chapel Hill, North Carolina, United States

## Abstract

Despite the significant burden of diabetes within African American and Black immigrant communities, there remains a notable gap in understanding the unique caregiving experiences and needs specific to these populations. Familial caregivers play a tremendous role in supporting disease management for their older relatives diagnosed with diabetes. The caregivers have a significant responsibility in providing instrumental (e.g., blood glucose monitoring, insulin administration) and material support (e.g., financial support, shopping) to older diabetic relatives. Research has shown that support from the family caregiver can improve the quality of life of a patient diagnosed with diabetes. This study aimed to investigate and elucidate the characteristics of family caregivers providing care to older diabetic relatives within African American and Black immigrant communities. With support from a librarian, we conducted a scoping review on PubMed, CINAHL, and Scopus databases. The review yielded 538 unique articles that were independently screened by two reviewers in Covidence. The preliminary data shed light on the characteristics of caregivers and the various roles they undertake in providing care. It highlights the challenges family caregivers face in these communities, including financial strain, limited access to resources, and cultural barriers. Additionally, the data reveal the specific support needs of these caregivers, indicating areas where interventions and support programs could be targeted to address their unique circumstances effectively. Our study serves as a foundation for further exploration and the development of targeted interventions to better support family caregivers in African American and Black immigrant communities.

